# Antitumor Activity of Death Receptor 5‑Targeted Camptothecin-Loaded Nanoparticles in Murine Syngeneic Models

**DOI:** 10.1021/acs.biomac.5c01884

**Published:** 2025-12-10

**Authors:** Anna J Boland, Michelle K Greene, Úna M Herron, Michael C Johnston, Peter Smyth, Hideo Yagita, Daniel B Longley, Christopher J Scott

**Affiliations:** 1 The Patrick G Johnston Centre for Cancer Research, School of Medicine, Dentistry and Biomedical Sciences, Queen’s University Belfast, 97 Lisburn Road, Belfast BT9 7AE, United Kingdom; 2 Future Medicines Institute, Queen’s University Belfast, 97 Lisburn Road, Belfast BT9 7AE, United Kingdom; 3 Juntendo University School of Medicine, Bunkyo-ku 113-8421, Tokyo, Japan

## Abstract

Death receptor 5 (DR5) is a key mediator of the extrinsic apoptotic pathway that is often upregulated in tumors, rendering it an attractive target for cancer therapy. Activation of DR5 requires oligomerization, which can be achieved through multivalent presentation of DR5 ligands on nanoparticles. DR5-targeted nanoparticles can efficiently agonize DR5 to inhibit the growth of human xenografts, although it remains unclear whether these effects would translate to a syngeneic tumor model with an immunocompetent microenvironment. Here, we develop camptothecin-loaded polymeric nanoparticles coated with the murine DR5 antibody MD5–1 and demonstrate their pro-apoptotic effects in murine cell lines *in vitro*. Moreover, we show that these nanoparticles inhibit the growth of MC38 colorectal allografts *in vivo* by >90% relative to control nanoparticles. Collectively, our work confirms that the antitumor efficacy of DR5-targeted nanoparticles extends to syngeneic models, paving the way for future studies to explore their impact on tumor immunity and the surrounding microenvironment.

## Graphical abstract

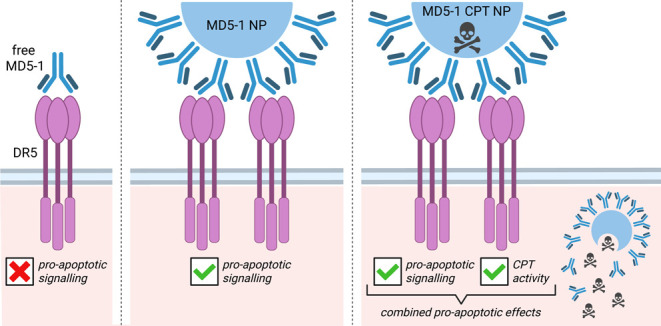

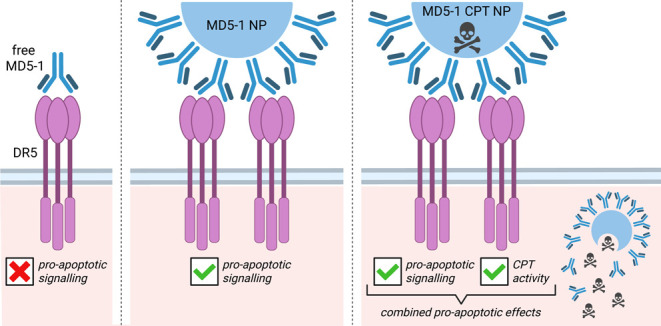

## Introduction

Colorectal cancer (CRC) poses a significant healthcare burden worldwide, ranking as the second and third leading cause of cancer-related mortality and incidence, respectively.^1^ Current management of CRC partly consists of conventional chemotherapy with agents such as 5-fluorouracil, irinotecan, oxaliplatin and capecitabine that present many challenges.^2^ These include poor stability, suboptimal pharmacokinetics and undesirable toxicities due to indiscriminate targeting of normal tissue. Treatment success and patient quality of life are impacted as a result, highlighting the need for more effective CRC treatment approaches.

Nanomedicine approaches offer a route to enhanced therapeutic windows for many anticancer agents.^3^ Nanoparticle-based vehicles formulated from materials such as lipids, polymers and metals are being evaluated to accommodate a variety of payloads. Many advantages are conferred through nanoencapsulation, such as enhanced circulation half-life and protection against the degradative action of enzymes and proteases.^4–6^ Moreover, nanoencapsulation can alleviate off-target effects, as exemplified by the reduced cardiotoxicity seen with liposomal doxorubicin (Doxil).^7^ Since the development of Doxil, nanomedicine has continued to rapidly gain momentum in cancer therapy, with many other formulations having received marketing authorization or currently undergoing (pre)­clinical evaluation.^8,9^

Nanoparticulate carriers can confer a passive targeting mechanism to their payloads due to their ability to accumulate within solid tumors via the ‘enhanced permeability and retention’ (EPR) effect.^10^ This arises owing to the ‘leaky’ vasculature and impaired lymphatic drainage within tumor tissue, which facilitate the infiltration and retention of nanoparticles. Moreover, intratumoral retention of passively targeted nanoparticles may be further enhanced via ‘active’ targeting. This involves the coupling of ligands to the nanoparticle surface, which engage cognate receptors typically expressed on the surface of tumor cells.^11^ A variety of ligands have been exploited in this context, in particular antibodies.^12^ Actively targeted nanoparticles offer distinct benefits since their recognition by cognate receptors often leads to endocytosis, enabling entrapped payloads to be delivered intracellularly in a ‘trojan horse’ approach. For example, we have previously shown that tumor deposition of the topoisomerase I inhibitor camptothecin (CPT) can be markedly improved following entrapment within actively targeted, versus passively targeted nanoparticles.^13,14^ However, despite this potential for enhanced drug delivery via passive and active targeting approaches, their effectiveness can vary due to factors such as heterogeneous vascularisation and receptor expression throughout tumors.

Death receptor 5 (DR5), a member of the tumor necrosis factor (TNF) receptor superfamily, is frequently upregulated in cancer and represents an attractive cell surface target.^15,16^ Importantly, binding and cross-linking of DR5 can also impart therapeutic effects through the activation of caspase-mediated cell death.^17^ Normally, DR5 is activated via binding to its endogenous ligand TRAIL, which is presented in a trimeric conformation.^18^ This leads to oligomerization of DR5, triggering a caspase-mediated signaling cascade that results in cell death via the extrinsic apoptotic pathway.^19^ Many mono- and bivalent DR5 agonists (e.g., recombinant TRAIL derivatives and monoclonal antibodies) have been trialled in the clinic without success, most likely due to their inability to induce higher order clustering of DR5.^20^ An alternative approach is to enhance the valency of DR5 agonists, for example through conjugation to nanoparticles.^21^

In previous work, we developed polymeric nanoparticles with a surface coating of the human DR5 antibody AMG 655 and demonstrated their superior antitumor effects versus the antibody in free format using human xenografts.^14,22^ Despite these promising outcomes, there remains a need to investigate how these effects translate to different DR5 agonists and in more complete and syngeneic tumor microenvironments. Several murine DR5 antibodies have been developed to date, including MD5–1 that has shown promising effects in preclinical models as a free agent,^23^ but has not yet been evaluated in nanoconjugated format.

In this current work, we aimed to address a key gap in the literature by evaluating the efficacy of nanoparticle-enabled multimerization of DR5 in a fully immunocompetent setting. To achieve this, we developed a multivalent MD5–1 platform through conjugation of the antibody to the surface of PEGylated PLGA nanoparticles. We compared the ability of the nanoparticles to target murine DR5 and induce apoptotic cell death in a panel of tumor cell lines, versus MD5–1 in native bivalent format, and whether these effects could be further enhanced through encapsulating CPT to yield nanoparticles with dual functionality. Moreover, we also investigated the *in vivo* tolerability and efficacy of the nanoparticles in the syngeneic MC38 model of CRC.

## Methods

### Cell Lines

MC38 and 3LL cells were cultured in Dulbecco’s Modified Eagle Medium (DMEM) (Gibco) supplemented with 10% fetal bovine serum (Gibco), 50 units/mL penicillin and 50 μg/mL streptomycin (Gibco). PAN02 cells were cultured in Roswell Park Memorial Institute (RPMI) 1640 medium (Gibco) supplemented with 10% fetal bovine serum, 50 units/mL penicillin and 50 μg/mL streptomycin. All cell lines were maintained in 5% CO_2_ in a humidified incubator at 37 °C.

### Nanoparticle Formulation and Antibody Conjugation

Nanoparticles were synthesized via a single emulsion-solvent evaporation method, whereby 15 mg PLGA RG502H (Sigma) and 5 mg PLGA-PEG-NHS (PolySciTech) were initially dissolved in 1 mL dichloromethane (DCM) to form the organic phase. In the case of drug-loaded nanoparticles, CPT (Sigma) was dissolved at 10 mg/mL in dimethyl sulfoxide (DMSO) and 10 μL (for *in vitro* studies) or 30 μL (for *in vivo* studies) was added to the organic phase. A 26-gauge needle attached to a 1 mL syringe was then used to inject the organic phase dropwise into an aqueous phase comprised of 7 mL 2.5% w/v poly­(vinyl alcohol) in 50 mM MES hydrate buffer at pH 5, under constant stirring at 600 rpm to form an emulsion. The emulsion was sonicated on ice in pulse mode (3 s on, 2 s off, for a total of 90 s) using a Model 120 sonic dismembrator (Fisher Scientific) set at an amplitude of 50% and left stirring at 600 rpm overnight at room temperature to enable DCM evaporation. Nanoparticles were subsequently washed via centrifugation x 3 (20 min, 16000 *g*, 4 °C) and resuspended at 1 mg polymer/mL in 50 mM MES hydrate buffer at pH 5. MD5–1 antibody (kindly gifted by Hideo Yagita, Juntendo University, Japan) conjugation was then achieved by adding 50 μg MD5–1/mg polymer to the nanoparticle suspension, followed by stirring at 90 rpm for 2 h at room temperature. Antibody-functionalized nanoparticles were finally washed via centrifugation (20 min, 16000 *g*, 4 °C) and resuspended in PBS for downstream cell-based studies.

### Nanoparticle Characterization

Nanoparticles were resuspended at 0.1 mg polymer/mL in distilled water and physicochemical characteristics including size, polydispersity index (PDI) and zeta potential were assessed using a NanoBrook Omni (Brookhaven Instruments Corporation) or a NanoSight NS300 (Malvern Panalytical). MD5–1 conjugation to nanoparticles was quantified using a Micro BCA protein assay kit (Thermo Fisher Scientific) as per the manufacturer’s instructions with some minor modifications. Briefly, standards were prepared by spiking known amounts of MD5–1 into either BLK NUDE NP or CPT NUDE NP suspended in PBS. Samples were also suspended in PBS and antibody content was interpolated from the standard curve. To quantify CPT entrapment, standards were prepared by spiking known amounts of CPT into BLK NUDE NP lysed in a 1:1 mixture of acetonitrile (ACN):DMSO at 0.5 mg polymer/mL. Samples were also lysed in 1:1 ACN:DMSO at 0.5 mg polymer/mL and CPT entrapment was interpolated from the standard curve following measurement of fluorescence at excitation and emission wavelengths of 330 and 460 nm, respectively. For transmission electron microscopy (TEM), nanoparticle formulations were resuspended at 2 mg polymer/mL in distilled water. Nanoparticle suspension (10 μL) was then added to Formvar/carbon support film copper grids (F196/050 TAAB). After approximately 15 min, grids were gently washed in distilled water before drying on filter paper. Stained samples were imaged using a JEOL JEM 1400plus transmission electron microscope operating at a HT voltage of 120 kV.

### Cell Viability Assays

Cell viability was assessed using the CellTiter-Glo assay (Promega) as per the manufacturer’s instructions with some minor modifications. Briefly, cells were seeded on white 96-well plates and left to adhere overnight. The culture media was then refreshed and cells were treated with nanoformulations or free MD5–1. At 72 h following treatment, spent media was removed to leave 25 μL per well, which was combined with 25 μL CellTiter-Glo Reagent. The plate was placed on a shaker for 10 min in darkness to facilitate cell lysis. Luminescence was then measured and viability was calculated relative to untreated cells. Caspase levels were also assessed as a readout of cell viability using the Caspase-Glo 3/7 assay (Promega) as per the manufacturer’s instructions with some minor modifications. Briefly, assays were performed as described above, except cells were treated for 24 h, Caspase-Glo Reagent was used in place of CellTiter-Glo Reagent and shaking was performed for 40 min. A further cell viability readout included Annexin V/PI analysis, where cells were seeded at 2 × 10^5^ per well on 6-well plates, left to adhere overnight and subsequently treated with nanoformulations. At 24 h following treatment, cells were trypsinised, centrifuged (5 min, 500 *g*, room temperature) and resuspended in 300 μL 1X Annexin V Binding Buffer (BD Pharmingen) containing 3 μL Annexin V FITC (BD Pharmingen). Following incubation for 15 min at room temperature, 2 μL of 1 mg/mL propidium iodide (Sigma) was added and cells were immediately analyzed on an Accuri C6 Plus flow cytometer (BD Biosciences). Data analysis was performed using FlowJo software (version 10) and necrotic, early apoptotic and late apoptotic cells were discriminated as PI-positive/Annexin V-negative, PI-negative/Annexin V-positive and PI-positive/Annexin V-positive populations, respectively.

### Western Blotting

Cells were seeded as required and left to adhere overnight. Culture media was then refreshed and cells were treated with nanoformulations, free MD5–1 or CPT. At 24 h following treatment, cells were detached by scraping and transferred alongside culture media to a 15 mL tube. Following centrifugation (5 min, 600 *g*, 4 °C), supernatants were aspirated and cell pellets were resuspended in PBS prior to a further centrifugation step as before. Supernatants were again aspirated and cells were lysed in RIPA buffer supplemented with protease inhibitor cocktail (Millipore) for 60 min on ice in 1.5 mL tubes. Lysates were then centrifuged (20 min, 17000 *g*, 4 °C) and supernatants were collected for immunoblotting while pellets containing debris were discarded. Sample protein content was quantified using the BCA protein assay kit (Thermo Scientific) as per the manufacturer’s instructions and 20–30 μg of each sample was denatured for 5 min at 95 °C in Laemmli buffer and subjected to SDS-PAGE until desired separation was achieved. Proteins were transferred onto a nitrocellulose membrane, which was then blocked in 5% milk powder in PBS/0.1% Tween 20 (PBST) for 1 h at room temperature. The membrane was probed with anti-FLIP (Cell Signaling Technology #56343, 1:2000), BAX (Cell Signaling Technology #5023, 1:2000), Bcl-2 (Cell Signaling Technology #2764, 1:2000), FADD (BD Biosciences #556402, 1:2000), procaspase-3 (Cell Signaling Technology #9662S, 1:2000), cleaved caspase-3 (Cell Signaling Technology #9661S, 1:1000), PARP (Cell Signaling Technology #9542, 1:2000) or β-actin (Sigma #A5316, 1:10,000) primary antibodies overnight at 4 °C, washed in PBST (3 × 10 min) and subsequently incubated with HRP-conjugated antimouse IgG (Cell Signaling Technology #7076) or antirabbit IgG (Cell Signaling Technology #7074) secondary antibodies for 1 h at room temperature. After a further wash in PBST as before, the membrane was covered in Western Lightning Plus-ECL substrate (PerkinElmer) and imaged using a G:BOX Chemi XX6 gel doc system (Syngene) equipped with GeneSys software. Densitometry analysis was conducted via ImageJ software, where protein density was assessed in comparison to a loading control band.

### Cell Surface Staining of DR5 and Calreticulin

For assessment of DR5 surface expression, cells were seeded as required on 6-well plates and left to adhere overnight. Culture media was aspirated and cells were washed in PBS/5% fetal bovine serum (FACS buffer) and detached. After two centrifugation washes (5 min, 300 *g*, 4 °C), cells were resuspended in 100 μL FACS buffer containing PE-conjugated anti-DR5 (Thermo #12–9908–42, 1:100) or isotype control (Thermo #12–4714–82, 1:200) antibodies and incubated for 30 min on ice in darkness. Cells were then washed as before and resuspended in PBS prior to analysis of fluorescence on an Accuri C6 Plus flow cytometer (BD Biosciences). For assessment of calreticulin surface expression, cells were seeded as required on 6-well plates and left to adhere overnight. Culture media was then refreshed and cells were treated with nanoformulations. At 24 h following treatment, cells were detached and transferred alongside culture media to a 15 mL tube. Following centrifugation (5 min, 300 *g*, 4 °C), supernatants were aspirated and cell pellets were resuspended in FACS buffer prior to a further centrifugation step as before. Supernatants were again aspirated and cells were resuspended in 100 μL PBS containing anticalreticulin (Thermo #PA3–900, 1:100) antibody and incubated for 30 min on ice. Cells were then washed as before, resuspended in 100 μL PBS containing Alexa Fluor 633-conjugated antirabbit IgG (Thermo #A-21070, 1:200) antibody and incubated for 30 min on ice in darkness. Cells were again washed as before and resuspended in PBS prior to analysis of fluorescence on an Accuri C6 Plus flow cytometer (BD Biosciences). All flow cytometry data analysis was performed using FlowJo software (version 10).

### *In Vivo* Studies

MC38 cells were resuspended at 2.5 × 10^6^/mL in a 1:1 mixture of PBS and Matrigel growth factor reduced basement membrane matrix (Corning) and 200 μL (5 × 10^5^ cells) was subcutaneously injected into one flank of 8-week old male C57BL/6 mice under gaseous isoflurane anesthesia. Animals were randomly assigned to receive various nanoformulations, which were resuspended at 40 mg polymer/mL in PBS. Once tumors reached an average volume of 100 mm^3^, 100 μL (4 mg polymer) of each nanoformulation was intravenously injected on days 7, 9, and 11. For CPT-loaded nanoformulations, a 4 mg polymer dosage equated to 1.36 ± 0.08 mg/kg CPT. Body weights and tumor volumes were monitored throughout the study, with the latter assessed using digital callipers and calculated as follows: (width^2^ x length) x 0.5. All animal experimentation was conducted under the authority of Project License PPL2875 granted by the Northern Ireland Department of Health. Studies were approved by the Animal Welfare and Ethical Review Body (AWERB) of Queen’s University Belfast.

### Data Analysis

Data were graphed and statistically analyzed using GraphPad Prism software. Statistical significance was determined via Student’s *t* test, one-way/two-way ANOVA with Tukey’s multiple comparisons test or Kruskal–Wallis test with Dunn’s multiple comparisons test as appropriate. A p value of <0.05 was considered statistically significant.

## Results

### Formulation of MD5–1-Functionalized Polymeric Nanoparticles

To construct nanoparticles displaying the murine DR5 antibody MD5–1, we employed a single emulsion-solvent evaporation approach with a polymer blend of PLGA 502H and PLGA-PEG-NHS. Our polymer choice of PLGA was in part due to its use in FDA-approved formulations, and also due to its potential for modification and for loading with a wide variety of drug candidates. PEG was incorporated to enhance the hydrophilicity and *in vivo* half-life of the nanoformulation, while the NHS moiety enabled facile, one-step conjugation of the nanoparticles to amine residues distributed throughout MD5–1. Surface functionalization of nanoparticles with MD5–1 did not significantly impact their size, with nontargeted nude nanoparticles (BLK NUDE NP) and antibody-conjugated nanoparticles (MD5–1 BLK NP) measuring 233 nm ± 7.2 and 238 nm ± 8.4 nm, respectively, via dynamic light scattering analysis (Figure 1A). This consistency in size between both nanoformulations was further supported by nanoparticle tracking analysis (Figure 1B). Moreover, BLK NUDE NP and MD5–1 BLK NP were highly monodisperse as indicated by low PDI values of 0.134 ± 0.04 and 0.115 ± 0.02 (Figure 1C), respectively, and TEM analysis provided further confirmation of the uniform morphology of both nanoformulations (Figure 1D). Zeta potential was also assessed via phase analysis light scattering, yielding similar values of −15.2 ± 0.9 mV for BLK NUDE NP and −8.9 ± 1.3 mV for MD5–1 BLK NP (Figure 1E).

**Figure 1:**
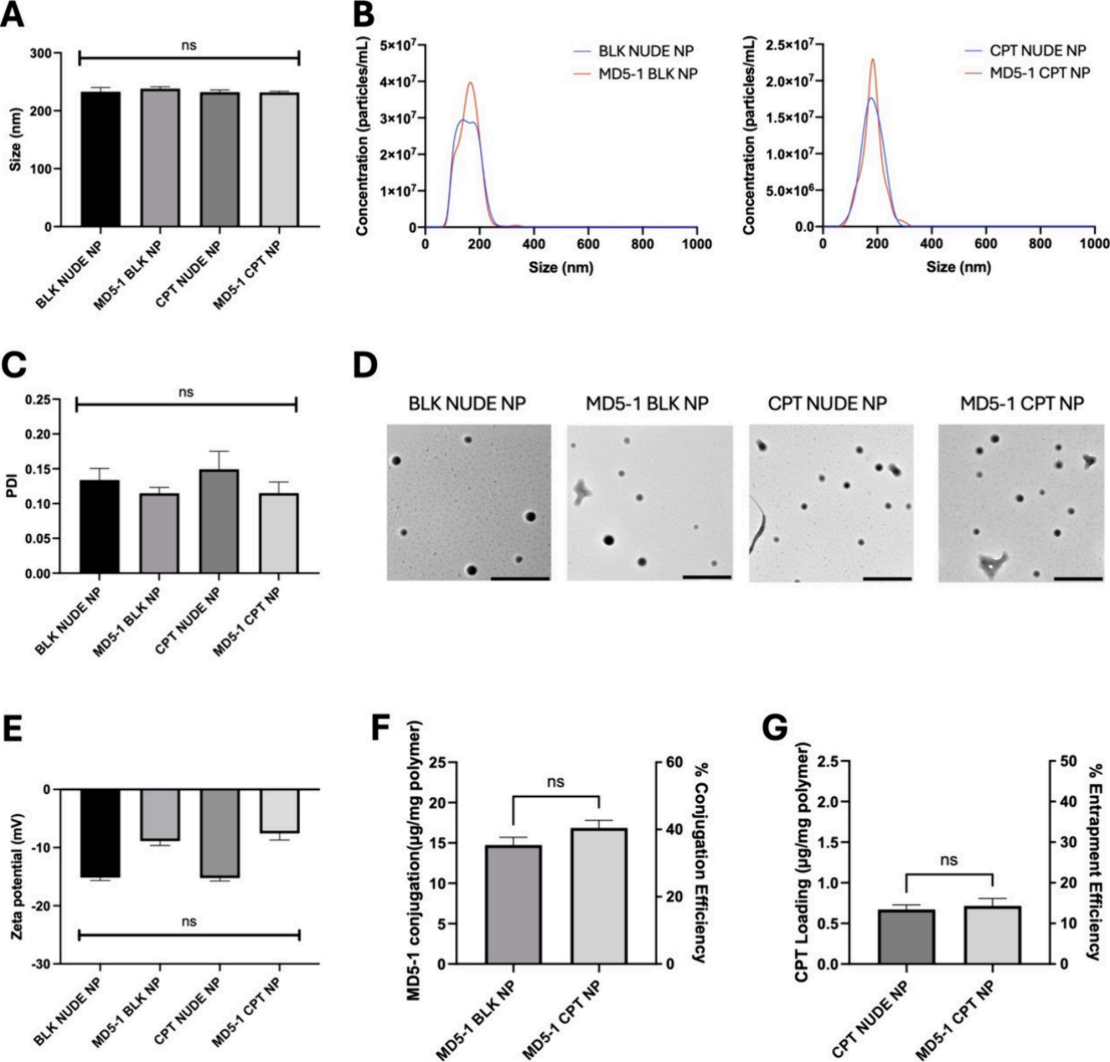
Physicochemical characterization of nanoparticle formulations. (A) Nanoparticle size measured via dynamic light scattering (*n* = 3 ± SEM) or (B) nanoparticle tracking analysis (representative of *n* = 2). (C) Nanoparticle polydispersity index (*n* = 3 ± SEM). (D) Transmission electron microscopy of nanoformulations (scale bar 500 nm). (E) Nanoparticle zeta potential measured via phase analysis light scattering (*n* = 3 ± SEM). (F) Quantification of MD5–1 conjugation to nanoparticles (*n* = 3 ± SEM). (G) Quantification of CPT loading within nanoparticles (*n* = 3 ± SEM).

### MD5–1 BLK NP Induce Cell Death via the Extrinsic Apoptotic Pathway

Having successfully developed MD5–1 BLK NP, their ability to induce apoptosis in murine cell line models was next investigated. Colorectal carcinoma MC38, lung carcinoma 3LL and pancreatic carcinoma PAN02 cell lines were chosen as suitable models for these studies given their variable expression levels of DR5 (Figure 2A) and other extrinsic apoptotic proteins, including the key inhibitory protein FLIP (Figure 2B). Treatment with the highest concentration of MD5–1 BLK NP led to reductions in viability of MC38 and 3LL cells by approximately 25% and 50%, respectively, whereas viability of PAN02 cells was unaffected (Figure 2C). Moreover, no reduction in viability was observed following treatment with either free MD5–1 or BLK NUDE NP in any of the cell lines. This suggests that multivalent presentation of MD5–1, enabled through conjugation to nanoparticles, is the key driving factor behind the observed reductions in cell viability. Interestingly, the level of DR5 (Figure 2A) and FLIP (Figure 2B) expression did not correlate with the degree of cell death observed, as viability of the 3LL cell line (63% of cells displayed cell surface DR5) was reduced to a greater extent than the PAN02 cell line (90% of cells displayed cell surface DR5). However, PAN02 cells expressed high levels of Bcl-2 (Figure 2B); a known downstream inhibitor of DR5-induced apoptosis.^24^ Consistent with the cell viability data, Western blot analysis revealed that MD5–1 BLK NP treatment of MC38 and 3LL but not PAN02 cells led to caspase-3 activation and PARP cleavage, together with a decrease in FLIP expression, which are key markers of apoptosis (Figure 2D).

**Figure 2:**
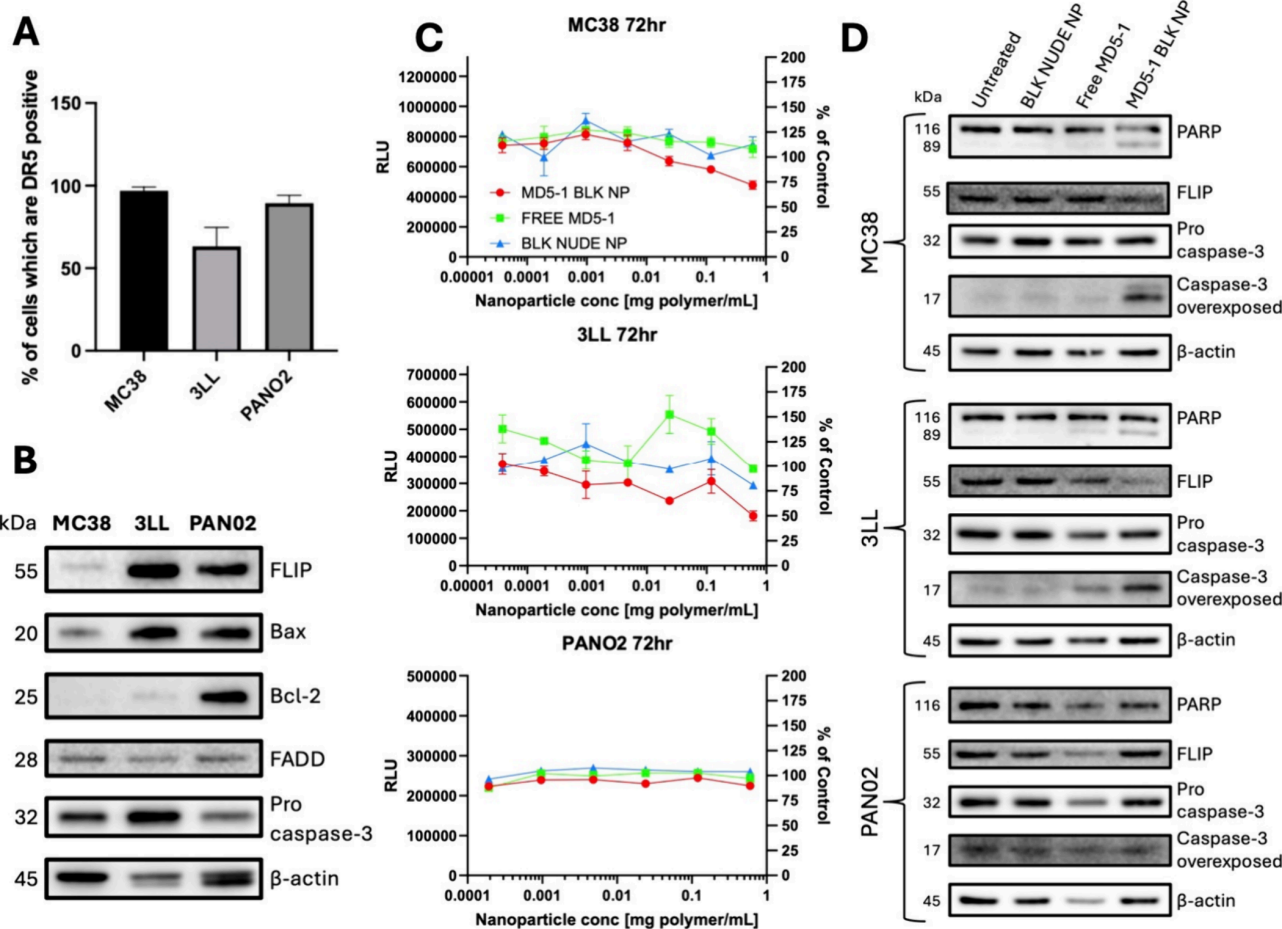
MD5–1 BLK NP induce apoptosis and caspase activation *in vitro*. (A) Cell surface expression of DR5 on murine cancer cell lines (*n* = 3 ± SEM). (B) Western blot analysis of the relative expression of apoptotic proteins in murine cancer cell lines (representative of *n* = 2). (C) Assessment of murine cancer cell viability using CellTiter-Glo following treatment with varying concentrations of MD5–1 BLK NP and controls for 72 h (representative of *n* = 3 ± SEM). (D) Western blot analysis of the relative expression of apoptotic proteins in murine cancer cell lines following treatment with MD5–1 BLK NP and controls (0.6 mg polymer/mL, 9 μg free MD5–1/mL) for 24 h (representative of *n* = 2). For both (C) and (D), free MD5–1 was added at an equivalent concentration to that provided by the corresponding MD5–1 BLK NP treatment.

### Incorporation of a CPT Payload within MD5–1 BLK NP Enhances Cell Death

To further enhance cell death induced by MD5–1 BLK NP, we next aimed to introduce a dual mode of action to these nanoparticles through entrapment of a cytotoxic cargo. Previously, we have shown that the topoisomerase I inhibitor CPT inhibits FLIP expression in human cancer cells, and this underpins the synergy observed between CPT and human anti-DR5 agonists.^14,22^ Here, we confirmed that this relationship was maintained in murine MC38 and 3LL cancer cells, where treatment with CPT concentrations of 0.1 μg/mL or higher led to downregulation of FLIP (Figure 3 **A and 3B**). These findings were not however replicated in PAN02 cells (Figure 3 **A and 3B**). Based on these data, CPT was chosen as a model drug for encapsulation within MD5–1 BLK NP.

**Figure 3:**
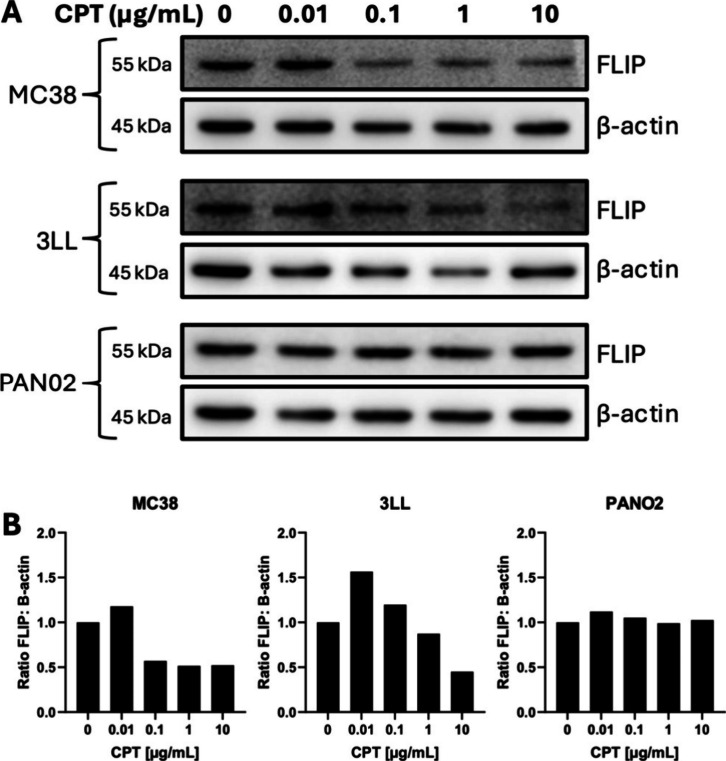
CPT inhibits FLIP expression in MC38 and 3LL cells, but not in PAN02 cells. (A) Western blot analysis of FLIP expression in murine cancer cell lines following treatment with increasing concentrations of CPT for 24 h (representative of *n* = 2). (B) Densitometry analysis of the ratio of FLIP to β-actin was performed using ImageJ software (representative of *n* = 2).

CPT was added to the organic phase during nanoparticle synthesis via the single emulsion-solvent evaporation method, leading to the successful development of CPT NUDE NP and MD5–1 CPT NP. Both nanoformulations were of comparable size, with a mean diameter of 232 nm ± 3.8 and 232 nm ± 2.0 nm, respectively, as measured by dynamic light scattering and further verified by nanoparticle tracking analysis (Figure 1 **A and 1B**). PDI values were also consistent between formulations, at 0.149 ± 0.057 for CPT NUDE NP and 0.115 ± 0.016 for MD5–1 CPT NP (Figure 1C), while TEM provided further confirmation of uniform morphology (Figure 1D). Moreover, zeta potential values were similar at −15.2 ± 0.9 mV for CPT NUDE NP and −7.6 ± 2.0 mV for MD5–1 CPT NP (Figure 1E). These size, PDI and zeta potential measurements were highly similar to those observed previously for BLK NUDE NP and MD5–1 BLK NP, with no significant differences apparent between the four nanoformulations. Moreover, MD5–1 conjugation was not impacted by CPT entrapment (Figure 1F), and CPT loading did not significantly differ between MD5–1-conjugated (0.72 ± 0.3 μg CPT/mg polymer, equating to an encapsulation efficiency of 14.3 ± 5.5%) and nude (0.67 ± 0.2 μg CPT/mg polymer, equating to an encapsulation efficiency of 13.4 ± 3.4%) nanoformulations (Figure 1G).

Subsequently, the biological functionality of the nanoformulations was evaluated. CPT-loaded nanoparticles were capable of inducing cell death in all lines tested, confirming that drug activity was maintained following the nanoformulation process (Figure 4A). MD5–1 CPT NP displayed the greatest potency in MC38 and 3LL cultures, leading to a superior reduction in cell viability compared to CPT NUDE NP at a concentration of 25 μg polymer/mL. However, the PAN02 cell line showed no increased sensitivity to MD5–1 CPT NP versus CPT NUDE NP.

**Figure 4:**
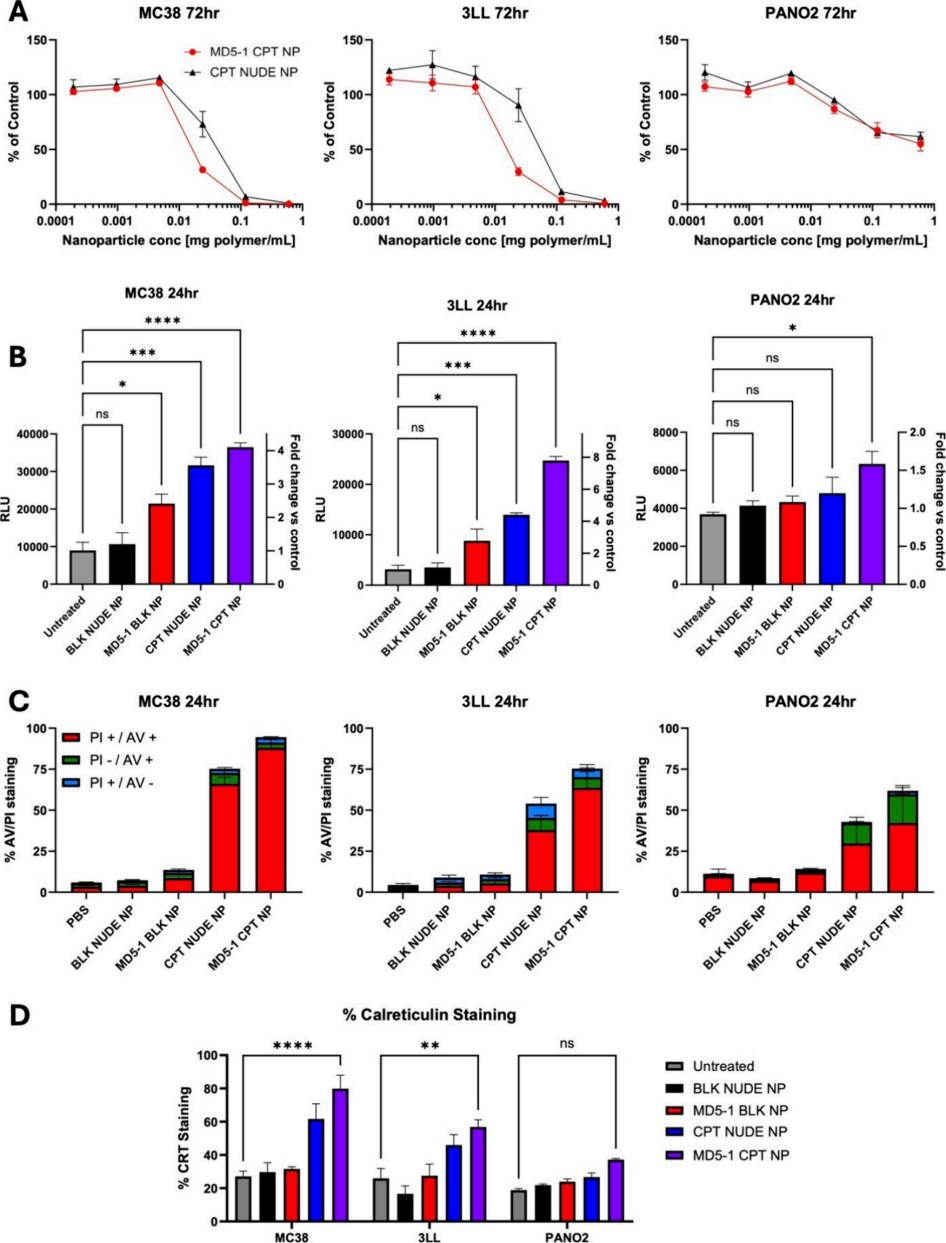
MD5–1 CPT NP drive a predominantly apoptotic phenotype, with some evidence of the potential induction of immunogenic cell death. (A) Assessment of murine cancer cell viability using CellTiter-Glo following treatment with varying concentrations of MD5–1 CPT NP and CPT NUDE NP for 72 h (representative of *n* = 3 ± SEM). (B) Caspase-Glo analysis of caspase 3/7 activation in murine cancer cell lines following treatment with MD5–1 CPT NP and controls (0.6 mg/mL polymer) for 24 h (representative of *n* = 3 ± SEM, *p < 0.05, ***p < 0.001, ****p < 0.0001, as measured by one-way ANOVA with Tukey’s multiple comparisons test). (C) Annexin V/PI analysis of apoptosis in murine cancer cell lines following treatment with MD5–1 CPT NP and controls (0.6 mg/mL polymer) for 24 h (representative of *n* = 3 ± SEM). (D) Cell surface expression of calreticulin on murine cancer cell lines following treatment with MD5–1 CPT NP and controls (0.6 mg/mL polymer) for 24 h (representative of *n* = 3 ± SEM, **p < 0.01, ****p < 0.0001, as measured by two-way ANOVA with Tukey’s multiple comparisons test).

In studies investigating the mechanism of cell death, both CPT NUDE NP and MD5–1 CPT NP led to enhanced activation of the apoptotic marker caspase 3/7 in MC38 and 3LL cells, which was more pronounced with the latter treatment (Figure 4B). Caspase 3/7 activation was less prominent in PAN02 cells and was only induced upon treatment with MD5–1 CPT NP. These findings were complemented by Annexin V/PI analysis, where treatment with CPT NUDE NP and MD5–1 CPT NP led to a marked increase in an apoptotic phenotype in all cell lines (Figure 4C). MC38 and 3LL cells showed the highest levels of late-stage apoptosis (PI-positive/Annexin V-positive) following MD5–1 CPT NP treatment, whereas levels were relatively lower in PAN02 cells. Instead, a large fraction of PAN02 cells remained in early stage apoptosis (PI-negative/Annexin V-positive), consistent with previous data showing that these cells possess a more resistant phenotype. Taken together, these findings indicate that MD5–1 CPT NP induce cell death, at least in part, via apoptosis.

Interestingly, while investigating alternative pathways of cell death, we discovered that MD5–1 CPT NP significantly upregulated the surface expression of calreticulin in MC38 and 3LL cells, with a similar trend observed in PAN02 cells despite not reaching significance (Figure 4D). Calreticulin is a key damage-associated molecular pattern (DAMP) involved in immunogenic cell death (ICD),^25^ highlighting the potential of MD5–1 CPT NP to drive cell death via differing mechanisms.

### *In Vivo* Assessment of MD5–1 CPT NP

Following the successful development and *in vitro* validation of MD5–1 CPT NP, we next sought to demonstrate their efficacy *in vivo*. For these studies, the MC38 colorectal model was employed given that these cells were highly sensitive to MD5–1 CPT NP *in vitro*. Treatment with MD5–1 BLK NP, CPT NUDE NP and MD5–1 CPT NP led to tumor regression versus the BLK NUDE NP control group, with the greatest inhibition seen in the MD5–1 CPT NP group on day 14 (Figure 5A). Tumor regrowth was apparent following treatment cessation, although for the MD5–1 CPT NP group, this occurred at a much slower rate and still remained below the initial day 6 tumor measurement at study termination. Furthermore, body weights did not decline throughout the study, suggesting that all nanoparticle formulations were well tolerated (Figure 5B). Taken together, these findings confirm that the efficacy of MD5–1 CPT NP translates to the *in vivo* setting.

**Figure 5:**
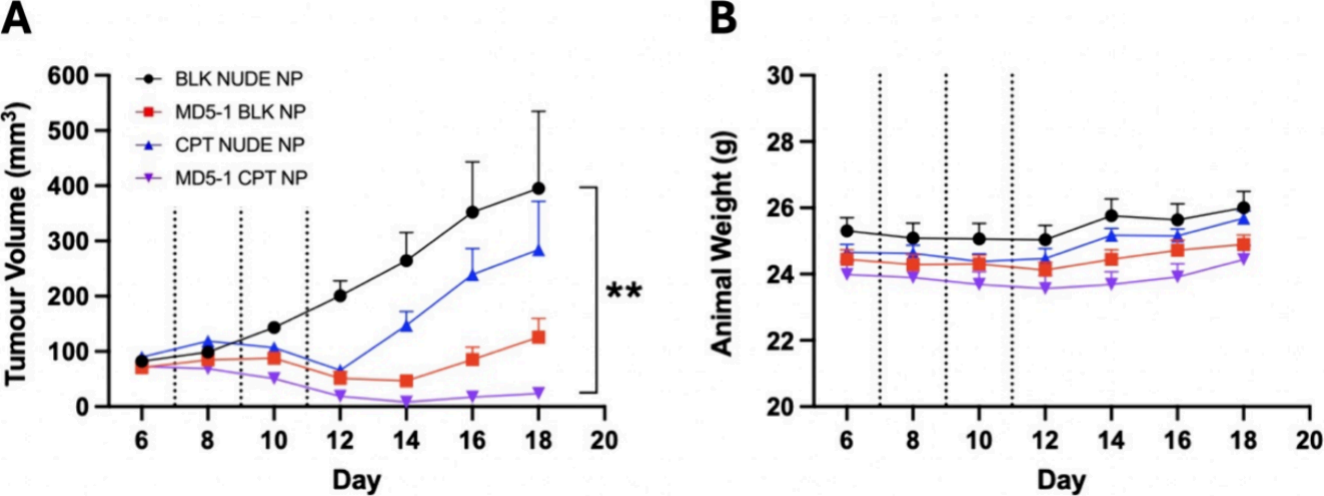
Tumor volume and body weight assessment during and after treatment of MC38 syngeneic tumors with MD5–1 CPT NP and controls. (A) Tumor volume measurements following treatment of subcutaneously implanted MC38 allografts with MD5–1 CPT NP or controls. Treatments consisted of 4 mg nanoparticles, which were delivered intravenously via tail vein injection on days 7, 9, and 11 as indicated by dashed lines (*n* = 8–9 per group ± SEM, **p < 0.01, as measured by Kruskal–Wallis test with Dunn’s multiple comparisons test). (B) Body weight monitoring throughout duration of study (*n* = 8–9 per group ± SEM).

## Discussion

In this work we have developed a MD5–1 conjugated nanoparticle with the topoisomerase 1 inhibitor CPT entrapped. This formulation was monodisperse and of consistent shape, drug entrapment and antibody conjugation. Conjugation of MD5–1 to the nanoparticle surface rendered it capable of inducing apoptosis in two out of three murine cancer cell lines and reducing tumor volume *in vivo*. Additionally, the entrapment of CPT increased the efficacy of the formulation while also increasing calreticulin presentation on the cell surface. This formulation was capable of producing a significant tumor volume reduction *in vivo.*

The conjugation of MD5–1 to the nanoparticle surface rendered it capable of inducing apoptosis where its free form did not. To the best of our knowledge, this is the first time this has been demonstrated with MD5–1 or indeed with any murine death receptor targeting moiety. This observation is consistent with human DR5 targeting therapies when conjugated to nanoparticles.^14,22,26,27^ This is also of note as antibodies alone have not exhibited clinically significant effects in clinical trials likely due to their lack of multivalency.^28^ While the need for multivalency to induce receptor clustering was known,^28^ it was hoped that the Fcγ receptor engagement of the antibody would help induce adequate receptor clustering at the disease site.^29^ However, Fcγ receptors are most often expressed on immune cells which may not be present in adequate number at the tumor site to produce efficacy. It was also found that a patient’s response could depend on their particular alleles of Fcγ receptor.^30^ There are other strategies to achieve multivalency which have reached clinical trial, however some of these trials have been terminated due to unexpected hepatotoxicity concerns.^31^ Further work is ongoing in an effort to finetune this multivalency to achieve optimal tumor apoptosis while leaving healthy tissue intact.^32^

The conjugation of a targeting moiety to the surface of a nanoparticle has been termed ‘active targeting’. To date, no nanoparticle formulation employing active targeting has been granted marketing authorization for use in humans. One such nanoparticle currently undergoing clinical testing is AVD-104 developed by Aviceda Therapeutics. At the time of writing it is in a phase 2 trial for the treatment of geographic atrophy secondary to age-related macular degeneration.^33,34^

Previously, we have shown that downregulation of FLIP can be synergistic for DR5 agonism in human models.^14,22^ In this work, we demonstrated the ability of CPT to partially downregulate FLIP in both MC38 and 3LL cell lines but not in the PAN02 line. This was found to be synergistic with nanoparticle bound MD5–1 in MC38 and 3LL cells. The additional resistance of the PAN02 line to MD5–1 CPT NP could be explained by its relatively high expression of the antiapoptotic protein Bcl-2 compared to MC38 and 3LL cells (Figure 2B). This suggests that the PAN02 line is already primed for resistance to apoptosis through the intrinsic apoptosis pathway. Future work could focus on the development of more potent FLIP inhibitors to elucidate whether complete downregulation of FLIP would allow for the direct cleavage of procaspase 3 by caspase 8 thereby circumventing the need for activation of the intrinsic apoptosis pathway.^19,35^

MD5–1 CPT NP were able to significantly reduce MC38 tumor volume *in vivo* with tumor volume not returning to baseline levels even 7 days after final treatment. Interestingly, MD5–1 BLK NP were more potent than CPT NUDE NP *in vivo* whereas the opposite trend was observed *in vitro*. This could be explained by the EPR effect where nanoparticles accumulate at the tumor site through leaky vasculature and fail to be cleared through a damaged lymphatic system.^36–38^ It could be hypothesized that the actively targeted MD5–1 BLK NP were able to bind to DR5 on cells at the tumor site aiding its accumulation relative to the passively targeted CPT NUDE NP.

In comparison to previous work where we tested a *human* DR5-targeted antibody in xenograft models, here we instead tested a *murine* DR5-targeted antibody in MC38 allografts.^14,22^ DR5 is known to be expressed systemically so MD5–1 NP could theoretically bind to death receptor in healthy tissues and cause agonism.^39^ This allograft model therefore offered the opportunity to test the systemic toxicity of a death receptor targeting antibody. Notably all mouse weights remained within acceptable limits for the duration of the study.

This immunocompetent mouse model was also chosen to best simulating how an intact immune system would act upon treatment with a DR5 targeted formulation. Previously our group has tested DR5 targeted therapies in human xenograft models in immunocompromised mice which does not elucidate any additional synergistic effect an intact immune system may offer.^14,22^ The presentation of the ‘eat me’ signal calreticulin on the surface of cells treated with CPT NUDE NP or MD5–1 CPT NP suggests that phagocytosis may come into play. It has previously been shown that an increase in calreticulin correlated with an increase in CD8+ T cell tumor infiltrate.^40^ Given these initial indications of immunomodulatory activity of our nanoparticles, it will be crucial to investigate this further in future studies, for example through immune profiling of tumors to identify the individual cell subsets present and their respective phenotypes. Moreover, a key priority moving forward will be to employ models whereby cell death mechanisms induced by our nanoparticles can be readily differentiated (e.g., classical versus immune-mediated cell death).

## Conclusions

In this work we developed a novel murine death receptor targeting nanoparticle with the topoisomerase 1 inhibitor CPT entrapped. This was capable of inducing a significant reduction in cell viability in two of the three cell lines tested and was also capable of causing a significant reduction of tumor volume *in vivo* while causing no toxicity. To the best of our knowledge, this is the first time a murine DR5 targeted antibody-based nanoparticle therapeutic has shown this efficacy in mouse models.

## Data Availability

All data requests should be directed to the corresponding author Christopher J Scott (c.scott@qub.ac.uk).
